# Quality of prescribing predicts hospitalisation in octogenarians: life and living in advanced age: a cohort study in New Zealand (LiLACS NZ)

**DOI:** 10.1186/s12877-019-1305-x

**Published:** 2019-12-19

**Authors:** Cristín Ryan, Ruth Teh, Simon Moyes, Tim Wilkinson, Martin Connolly, Anna Rolleston, Mere Kepa, Ngaire Kerse

**Affiliations:** 1 School of Pharmacy and Pharmaceutical Sciences, Trinity College Dublin, Dublin, Ireland; 2 Department of General Practice and Primary Health Care, Faculty of Medical and Health Sciences University of Auckland, Auckland, New Zealand; 3 Older People’s Health, University of Otago, Dunedin, New Zealand; 4 Freemasons Department of Geriatric Medicine, Faculty of Medical and Health Sciences University of Auckland, Auckland, New Zealand; 5 Te Kupenga Haoura Māori, Faculty of Medical and Health Sciences, University of Auckland, Auckland, New Zealand

**Keywords:** Older people, Ethnicity, Longitudinal study, Appropriate prescribing

## Abstract

**Background:**

Prescribing for older people is complex, and many studies have highlighted that appropriate prescribing in this cohort is not always achieved. However, the long-term effect of inappropriate prescribing on outcomes such as hospitalisation and mortality has not been demonstrated. The aim of this study was to determine the level of potentially inappropriate prescribing (PIP) for participants of the Life and Living in Advanced Age: A Cohort Study in New Zealand (LiLACS NZ) study at baseline and examine the association between PIP and hospitalisation and mortality at 12-months follow-up.

**Methods:**

PIP was determined using STOPP/START. STOPP identified potentially inappropriate medicines (PIMs) prescribed, START identified potential prescribing omissions (PPOs). STOPP/START were applied to all LiLACS NZ study participants, a longitudinal study of ageing, which includes 421 Māori aged 80–90 years and 516 non-Māori aged 85 years. Participants’ details (e.g. age, sex, living arrangements, socioeconomic status, physical functioning, medical conditions) were gathered by trained interviewers. Some participants completed a core questionnaire only, which did not include medications details. Medical conditions were established from a combination of self-report, review of hospital discharge and general practitioner records. Binary logistic regression, controlled for multiple potential confounders, was conducted to determine if either PIMs or PPOs were associated with hospital admissions and mortality (*p* < 0.05 was considered significant).

**Results:**

Full data were obtained for 267 Māori and 404 non-Māori. The mean age for Māori was 82.3(±2.6) years, and 84.6(±0.53) years for non-Māori. 247 potentially inappropriate medicines were identified, affecting 24.3% Māori and 28.0% non-Māori. PIMs were not associated with 12-month mortality or hospitalisation for either cohort (*p* > 0.05; adjusted models). 590 potential prescribing omissions were identified, affecting 58.1% Māori and 49.0% non-Māori. PPOs were associated with hospitalisation (*p* = 0.001 for Māori), but were not associated with risk of mortality (p > 0.05) for either cohort within the 12-month follow-up (adjusted models).

**Conclusion:**

PPOs were more common than PIMs and were associated with an increased risk of hospitalisation for Māori. This study highlights the importance of carefully considering all indicated medicines when deciding what to prescribe. Further follow-up is necessary to determine the long-term effects of PIP on mortality and hospitalisation.

## Background

It is widely acknowledged that the population is ageing. In 2013, 14% of the total population worldwide was aged over 80 years, and this is projected to rise to 19% by 2050, which will equate to 392 million persons [1]. With increasing age, there is an increase in healthcare resource utilisation from an increased prevalence of chronic conditions and their inherent treatment burdens [2, 3]. Prescribing of medicines to treat these conditions is one of the most common healthcare interventions that doctors undertake, an intervention which becomes increasingly complex in older people with multimorbidity [3, 4]. Increased risk of medication-related problems e.g. adverse drug reactions and drug interactions, increase challenges around prescribing, particularly as older people heterogeneously display altered pharmacokinetic and pharmacodynamic handling of medicines, compared with their younger counterparts [5, 6].

Several screening tools have been developed to address these challenges and improve prescribing practices for older people by preventing potentially inappropriate prescribing (PIP). PIP is defined as any instance of prescribing which is sub-optimal and increases the risk of harm to a patient, particularly when there is a safer, more effective alternative available [7]. One such screening tool is Screening Tool of Older Person’s Prescriptions and Screening Tool to Alert doctors to Right Treatment (STOPP/START), [8] originally published in 2008 and recently updated in 2015 (STOPP/STARTV2) [9]. STOPP identifies medicines that should not be prescribed (potentially inappropriate medicines (PIMs)), whilst START addresses errors of omissions i.e. the absence of a clinically indicated medicine, or potential prescribing omissions (PPOs) [8, 9]. STOPP/START was developed to target prescribing improvement initiatives for older people of all ages, and takes patients’ comorbid and clinical status into account. For example, START recommends the prescribing of statin therapy for patients who have a documented history of coronary, cerebral or peripheral vascular disease, if the patient’s functional status remains independent for actives of daily living and life expectancy is greater than five years [8]. In other words, statin therapy would not be recommended if the patient had a limited life expectancy. STOPP/START 2008 [8] has been used widely throughout a variety of European countries and in different patient settings to identify the level of PIP for older people [10–13]. Potentially inappropriate prescribing rates of up to 20% have been reported for older people residing in primary care, 58% for older people in the acute hospital setting and 70% for nursing home residents [10, 13]. STOPP/START 2008 [8] has good inter-rater reliability between pharmacists and physicians [14, 15]; in one study using STOPP in routine practice prevented adverse drug reaction related hospital admissions [16]; in another study using STOPP and START together improved older peoples’ overall level of medication appropriateness [17]. To date, there are no published studies using the STOPP/STARTV2.

Prescribing for indigenous and ethnic minority groups may pose further challenges as disparities in health are well documented, [18, 19] but this is largely unexplored in octogenarians. On the one hand, Māori (indigenous people of New Zealand) have shorter life expectancy and poorer health outcomes compared to non-Māori. Reasons for this may include a combination of distal effects of colonisation [20, 21] structural disparities in access to care, [22, 23] and racism within health services [23]. Furthermore, attitudes to medication taking differs amongst ethnic groups, and there are differences in therapeutic partnerships between prescribers and patients of varying ethnicity [24]. On the other hand, it is acknowledged that Māori care very much about their medication and are very involved in self-management [25]. Managing potential discrepancies between physicians and Māori world view may be necessary to improve prescribing and outcomes related to prescribing.

Studies that have investigated the occurrence of PIP using STOPP/START 2008 generally focus on cohorts involving people aged 65 years and older. So far, none of these studies have looked specifically at prescribing practices for those in advanced age (i.e. > 80 years), nor investigated prescribing for indigenous patient groups.

In New Zealand, a longitudinal study of ageing (LILACS NZ) has been underway since 2010 [26]. In this study, Māori (80–90 years) and non-Māori (> 85 years) are being followed up at yearly intervals to examine predictors of successful ageing and document disparities in advanced ag e[27, 28]. Medications and diagnoses are a main part of the health measures collected, and therefore this study aims to determine the level of appropriate prescribing using the STOPP/START criteria and whether those levels are related to hospitalisation and mortality over time.

### Aims

This study aimed to describe the classes of medicines routinely prescribed for Māori and non-Māori octogenarians, determine the level of PIP for these participants at baseline using the STOPP/STARTV1 criteria, and to determine the association between PIP (either as a PIM or a PPO) and hospitalisation and mortality at 12 months follow-up.

## Methods

### Data collection

Te Puāwaitanga O Ngā Tapuwae Kia Ora Tonu - Life and Living in Advanced Age: a Cohort Study in New Zealand (LiLACS NZ) is a cohort study of those in advanced age in New Zealand. Within a geographic boundary of the Bay of Plenty District Health Board and the Lakes District Health Board (excluding the Taupo region) all Māori born in 1920 to 1930 (80–90 years of age) and non-Māori born from 1925 (aged 85 years) were identified in 2010 using multiple overlapping sampling strategies including the electoral roll, primary health care databases, word of mouth, whanaungatanga (kin relations), tribal organisations, cultural networks, publicity and posters in residential care and general practices. Participants were invited by a person known to them, or their general practitioner and 927 were successfully enrolled in the cohort study (57% participation rate). Kaupapa Māori methods [29] were used to ensure engagement and assessment were appropriate for Māori and Te RōpuKaitiaki o Ngā Tikanga Māori (group of senior Māori tribal leaders) was convened to provide oversight and direction to ensure Māori protocols and practices were respected.

Written informed consent was obtained with appropriate translation of documents [30]. Data were gathered in face-to-face, standardised questionnaires by trained interviewers using standardised techniques in the person’s home, a research site or the local clinic depending on participant choice. Data collection included comprehensive information about socioeconomic status, health, function, quality of life and health services in a comprehensive interview. The development of the interview guide has been described elsewhere [28, 31]. Some participants completed a core questionnaire only which did not include the medication data.

### Measures

Age, sex, living arrangement, marital status, education, lifetime occupation were self-reported. New Zealand deprivation index 2006 (NZDep) measure of the level of socioeconomic deprivation in small geographic areas of New Zealand) was obtained from the address given at the time of first interview [32, 33]. Diagnoses were ascertained by self-report and verified by a GP record review completed with standardised techniques. Hospitalisation records were obtained from Ministry of Health national records by matching the participant’s unique National Health Index number and were also used to verify hospitalisations. Diagnoses were established from combinations of self-report validated against hospital and general practitioner records. Renal function was judged from calculation of an Estimated GFR (eGFR) using Modification of Diet in Renal Disease (MDRD) formula [34]. Methods of agreement for diagnoses have been described elsewhere [35].

The Geriatric Depression Scale (GDS) was used to establish depressive symptomatology (6) with established cut points for mild to moderate and severe depressive symptoms [36]. The Nottingham Extended Activities of Daily Living scale (NEADL) was used to establish functional status. This gives a score of 22 with higher function meaning greater independence [37]. The modified mini-mental state (3MS) examination was used to assess cognition. Patients were not excluded based on the outcome of this assessment.

In a standard interview, medications were examined by interviewers and recorded from the bottles and packets while verifying that the medication was taken. Thus medications, as taken, were recorded including “as required” medications and over-the-counter medications. Medications were then coded using the World Health Organisation’s Anatomical Therapeutic Chemical (ATC) Classification System to facilitate analysis [38].

Outcomes of all hospitalisations and mortality were ascertained by using the individual participant National Health Index (NHI) matched to routinely held New Zealand Ministry of Health data in administrative databases.

### STOPP/START

In total, there are 65 STOPP criteria and 22 START criteria in version 1. STOPP/STARTV2 was not published at the time when this study was conceived and when analyses was undertaken, therefore, the original STOPP/START criteria were used. As full clinical records were not accessible, we were not able to assess all prescribing rules within the STOPP criteria for each patient. All START criteria were applied to all patient records. Two criteria for the cardiovascular system were combined and assessed as one prescribing rule. These were “*warfarin in the presence of chronic atrial fibrillation*” and “*aspirin in the presence of chronic atrial fibrillation, where warfarin is contraindicated*”. We noted this as one criterion “*warfarin or aspirin in the presence of chronic atrial fibrillation*”. Twelve of the STOPP criteria were not used, due to lack of patient information available and are detailed in Table 1. All patients with available data involved in the LiLACS NZ were included in this study, including those who were not prescribed any medicines. This was to ascertain if they had any prescribing omissions as defined by START.

**Table 1:** STOPP criteria that were not assessed in the LiLACS NZ study

Criteria	Reason for non-assessment
Cardiovascular System	
Aspirin with a past history of peptic ulcer disease without histamine H_2_ receptor antagonist or Proton Pump Inhibitor	Peptic Ulcer Disease was recorded only if an active condition i.e. history was not investigated
Warfarin for first, uncomplicated deep venous thrombosis for longer than 6 months	Reason for treatment with warfarin was not documented
Warfarin for first uncomplicated pulmonary embolus for longer than 12 months duration	Reason for treatment with warfarin was not documented
Aspirin, clopidogrel, dipyridamole or warfarin with concurrent bleeding disorder	Presence of bleeding disorder was not recorded
Central Nervous System	
TCA’s with prostatism or prior history of urinary retention	History of urinary retention
Long-term (i.e. > 1 month) neuroleptics as long-term hypnotics	Indication for neuroleptics was not documented
Selective serotonin re-uptake inhibitors (SSRI’s) with a history of clinically significant hyponatraemia	Hyponatraemia was noted if present, but not if patients had a history of it
Gastro-intestinal System	
Diphenoxylate, loperamide or codeine phosphate for treatment of diarrhoea of unknown cause	Diarrhoea as an indication was not recorded
Diphenoxylate, loperamide or codeine phosphate for treatment of severe infective gastroenteritis	Gastroenteritis as an indication was not recorded
Musculoskeletal System	
Non-steroidal anti-inflammatory drug (NSAID) with history of peptic ulcer disease or gastrointestinal bleeding, unless with concurrent histamine H_2_ receptor antagonist, PPI or misoprostol	Peptic Ulcer Disease was recorded only if an active condition i.e. history was not investigated
Urogenital System	
Alpha-blockers in males with frequent incontinence i.e. one or more episodes of incontinence daily	Presence of incontinence was not recorded
Alpha-blockers with long-term urinary catheter in situ i.e. more than 2 months	Presence of a catheter was not recorded

### Statistical analysis

All data were imported into Statistical Package for the Social Sciences (SPSS) version 19.0 for analysis. Descriptive statistics were performed to describe the demographic profile of the participants. The Mann-Whitney U test for non-parametric data was conducted to compare the presence of potentially inappropriate prescribing between Māori and non-Māori participants (Table 2). Binary logistic regression was then conducted to determine if either PIMs or PPOs (both used as binary variables), as identified by STOPP and START respectively, were associated with hospital admissions, and mortality (unadjusted analyses presented in Additional file 2 Figure S2). Regression models were built to adjust for potential confounders including: age, gender, prior 12-month GP utilisation, socioeconomic deprivation, Congestive Heart Failure (CHF), number of medications and functional status (NEADL). These variables were identified from the literature and from univariate analyses as being predictive of hospitalisations in this sample (data not shown). A *p*-value of < 0.05 was considered significant in the final models.

**Table 2:** Demographics of the study population

Demographics	Māori Total (*n* = 267) N (%) or Mean (SD)	Māori with at least one PIM (*n* = 65) N (%) or Mean (SD)	Māori with at least one PPO (*n* = 155) N (%) or Mean (SD)	Non-Māori Total (*n* = 404) N (%) or Mean (SD)	Non-Māori with at least one PIM (*n* = 108) N (%) or Mean (SD)	Non-Māori with at least one PIM (*n* = 195) N (%) or Mean (SD)
Age (Mean ± SD)	82.3 (±2.6)	82.1(±2.6)	82.4(±2.7)	84.6 (±0.5)	84.6 (±0.5)	84.6 (±0.5)
Gender (Female)	160 (59.9)	32 (49.2)	96 (61.2)	214 (53.0)	61 (56.5)	112 (57.4)
Socioeconomic Deprivation Scores						
(NZDep) 1–4	37 (13.9)	9 (13.8)	23 (14.8)	101 (25.0)	23 (21.3)	48 (24.6)
5–7	65 (24.3)	13 (20.0)	34 (21.9)	171 (42.3)	45 (41.6)	83 (42.6)
8–10	165 (61.8)	19 (29.2)	43 (27.7)	132 (32.7)	45 (41.6)	67 (34.4)
CHF (Present)	81 (30.3)	37 (57.0)	63 (40.6)	79 (19.6)	34 (31.5)	53 (27.2)
Depressive symptoms (GDS)						
0–3	190 (74.5)	45 (69.2)	87 (56.2)	315 (80.4)	73 (67.6)	126 (64.6)
4–9 (moderate)	62 (24.3)	16 (24.6)	60 (38.7)	74 (19.1)	34 (31.5)	67 (34.4)
10+ severe	3 (1.2)	2 (3.1)	3 (1.9)	2 (0.5)	1 (92.6)	2 (1.0)
Functional Status in NEADL Mean ± SD	17.24 ± 4.58	16.7 (±4.4)	16.5 (±4.9)	17.64 ± 4.03	16.5 (±4.7)	16.9 (±4.4)
Hospitalisation in 12 month follow up	99 (40.4)	28 (43.1)	74 (47.7)	163 (41.0%)	51 (47.2)	88 (45.1)

KEY: *N* Number; % = percent; *SD* Standard Deviation

GDS Geriatric Depression Scale score, 0–15, higher score is more depressive symptoms

NEADL Nottingham Extended Activities of Daily Living score, 0–22, higher score is better function

The Northern X Regional Ethics Committee Ministry of Health New Zealand approved all aspects of the LiLACS study in 2009 (Ref: NTX/09/09/088). All participants provided written informed consent.

## Results

Full data were obtained for 267 Māori and 404 non-Māori who completed the comprehensive interview. Those who completed only the core interview (*n* = 261) were more likely to be in residential care (52 (20%) of core vs 23 (3%) of comprehensive; chi square 69.8; *p* < 0.001), more likely to be dependent in ADLs (46 (18%) of core dependent in getting in and out of bed vs 17 (3%) of comprehensive respondents Chi Square 68.5; *p* < 0.001) and more likely to be Māori (150 (36%) of Māori completed only the core vs 111 (22%) of non-Māori completed the core; Chi Square 26.3; *p* < 0.001).

### Demographics

The mean age for Māori was 82.3(±2.6) years, while that for non-Māori was 84.6(±0.53) years (Table 2). A higher proportion were female for both the Māori and non-Māori cohorts (59.9 and 53.0% respectively). Almost two thirds (61.8%) of the Māori cohort had deprivation scores of 8–10 (higher levels equating to higher deprivation) indicating residence in an area of high socioeconomic deprivation, while the corresponding deprivation for non-Māori cohort was 32.7%. A similar proportion in each group were hospitalised in the previous year. The functional status of each cohort was relatively high (17.24 ± 4.58 and 17.64 ± 4.03 for Māori and non-Māori respectively), indicating independence rather than dependence (Table 2).

### Medicines prescribed

The total number of medicines prescribed was 3222; 1987 (61.7%) prescribed for non-Māori participants and 1235 (38.3%) prescribed for Māori participants. The mean number of medicines prescribed per person was similar for Māori 4.63 (±3.24) and non-Māori 4.92 (±3.18).

According to the ATC codes assigned to each medicine, the highest proportion of medicines was prescribed for the Cardiovascular System conditions, followed by conditions of the Alimentary Tract and Metabolism, Blood and Blood forming organs and the Central Nervous System. A higher percentage of non-Māori participants were prescribed medicines for the central nervous system than Māori participants (Additional file 1 Figure S1). Overall, 126 (18.8%) died during the first year, and 262 (39.0%) were hospitalised.

### Overall potentially inappropriate prescribing (PIP)

The proportion of participants to have either a PIM or a PPO was similar for each cohort (Table 3), with 65.5% of Māori having either a PIM (identified by STOPP) or a PPO (identified by START), with the corresponding value for non-Māori being 62.1%. For both cohorts, there were more omissions than there were potentially inappropriate medicines.

**Table 3:** The prevalence of potentially inappropriate prescribing as defined by the STOPP/START criteria in the LiLACS NZ cohort

Demographics	Māori (*n* = 267)	Non-Māori (*n* = 404)	*P* Value
Mean number of medicines prescribed per patient (Range; ±SD)	4.63 (0–14; ±3.24)	4.92 (0–15; ±3.18)	0.288~
PIM STOPP n (%)	65 (24.34)	113 (27.97)	0.171^
PPO START n (%)	155 (58.1)	198 (49.0)	0.013^
PIM or PPO n (%)	175 (65.5)	251 (62.1)	0.207^

Key: *PIM* Potentially inappropriate medicine; *PPO* Potential prescribing omission; ~ = Mann-Whitney U; ^= Chi Square

### Potentially inappropriate medicine (PIMs) identified by STOPP

Overall, a total of 247 PIMs were identified using the STOPP criteria (Additional file 3). The commonest potentially inappropriate medications prescribed were high dose proton pump inhibitors for greater than 8 weeks for peptic ulcer disease. The prescribing of long-term opiates in those who are recurrent fallers was the second commonest, with the prescribing of aspirin to patients who do not have a history of coronary, cerebral or Peripheral Vascular Disease (PVD) symptom or an occlusive event being the third most common.

There were three of the 53 prescribing scenarios that were significantly more common in non-Māori participants than Māori. They were: the prescribing of tricyclic antidepressants in combination with an opiate or calcium channel blocker (*p* = 0.036); the prescribing of an NSAID in patients with moderate-severe hypertension (p = 0.036) and the prescribing of neuroleptics to patients who have a history of falling (*p* = 0.046).

### Potential prescribing omissions (PPOs) identified by START

A total of 590 PPOs were identified for this cohort (Additional file 4). The most common omission was the absence of prescribing of antidepressants in the presence of moderate/severe depressive symptoms (score of 5+ on the GDS), 12% amongst non-Māori and 19% amongst Māori.

The omission of β blockers in patients with chronic stable angina was the second most common omission, and the omission of Calcium and Vitamin D_3_ supplement in patients with known osteoporosis was the third. There were few differences between Māori and non-Māori in terms of the types of omissions identified.

The omission of aspirin or clopidogrel in those with a documented history of atherosclerotic coronary, cerebral or peripheral vascular disease (9.0% for Māori Vs 3.8% for non- Māori *p* < 0.001), and the omission of bisphosphonate in patients taking maintenance corticosteroid therapy (5.8% Vs 3.5%, *p* = 0.035) were more common in Māori participants.

Conversely, the omission of antihypertensives in patients with a systolic blood pressure of over 160 mmHg, omission of statin therapy in those with a documented history of atherosclerotic coronary, cerebral or peripheral vascular disease was more common in non-Māori (*p* = 0.018 and *p* = 0.009 respectively).

### PIMs and PPOs as predictors of hospitalisations in the first 12 months follow up

#### Potentially inappropriate medicines (PIMs)

The occurrence of a PIM alone in the Māori cohort was not significantly associated with 12 month mortality (*p* = 0.946) or 12 month hospitalisation (*p* = 0.619) when adjusted for age, gender, prior 12-month GP utilisation, socioeconomic deprivation, CHF, number of medications and functional status (NEADL) (Table 4). Likewise, in the non-Māori cohort, PIMs were not associated with 12-month mortality (*p* = 0.338) or 12-month hospitalisation (*p* = 0.371), when adjusted for gender, prior 12-month GP utilisation, deprivation, CHF, number of medication and NEADL.

**Table 4:** The association between the occurrence of PIMs and PPOs using STOPP and START respectively and 12 month mortality and hospitalisation for Māori and non- Māori participants

Māori Hosptialisation					
Participants with at least one instance of PIP		Any hospitalisation at 12 months follow-up n (column %)		Adjusted^a^	
				OR (95% CI)	*P* Value
PIMs	Yes (57)	Yes	No	1.20 (0.59, 2.44)	0.619
		28 (49.1)	29 (50.9)		
	No(189)	71 (37.6)	118 (62.4)		
PPOs	Yes(99)	74 (74.7)	25 (25.3)	2.80 (1.54, 5.10)	0.001
	No(147)	69 (46.9)	78 (53.1)		
PIMs or PPOs	Yes(159)	78 (49.1)	81 (50.9)	2.41 (1.26, 4.59)	0.008
	No(87)	21 (24.1)	66 (75.7)		
Māori Mortality					
Participants with at least one instance of PIP		Mortality at 12 months follow-up (*n* = 20) (%) n (column %)		Adjusted^a^	
				OR (95% CI)	*P* Value
PIMs	Yes (65)	Yes	No	1.04 (0.30, 3.70)	0.946
		6 (9.2)	59 (90.8)		
	No (202)	14 (6.9)	188 (93.1)		
PPOs	Yes (155)	18 (11.6)	137 (88.4)	3.61 (0.70, 18.56)	0.125
	No (112)	2 (1.8)	110 (98.2)		
PIMs or PPOs	Yes (175)	18 (10.3)	157 (89.7)	1.92 (0.36, 10.19)	0.445
	No (92)	2 (2.2)	90 (97.8)		
Non-Maori Hosptialisation					
Participants with at least one instance of PIP		Any hospitalisation at 12 months follow-up^aa^		Adjusted^a^	
				OR (95% CI)	*P* Value
PIMs	Yes (113)	Yes	No	1.25 (0.77, 2.02)	0.371
		51 (45.1)	62 (54.9)		
	No (285)	112 (39.3)	173 (60.7)		
PPOs	Yes (197)	88 (44.7)	109 (55.3)	1.44 (0.94, 2.20)	0.090
	No (201)	75 (37.3)	126 (62.7)		
PIMs or PPOs	Yes (163)	109 (66.9)	54 (33.1)	1.40 (0.90, 2.17)	0.141
	No (235)	141 (60.0)	94 (40.0)		
Non-Maori Mortality					
Patients with at least one instance of PIP		Mortality at 12 months follow-up (*n* = 18) (%)^aaa^		Adjusted^a^	
				OR (95% CI)	*P* Value
PIMs	Yes (113)	Yes	No	1.698 (0.575, 5.011)	0.338
		8 (7.1)	105 (92.9)		
	No (291)	10 (3.4)	281 (96.6)		
PPOs	Yes (198)	13 (6.6)	185 (93.4)	2.37 (0.71,7.86)	0.160
	No (206)	5 (2.4)	201 (97.6)		
PIMs or PPOs	Yes (251)	16 (6.4)	235 (93.6)	7.21 (0.90, 57.58)	0.062
	No (153)	2 (1.3)	151 (98.7)		

Key: PIP = Potentially Inappropriate Prescribing; PIMs = Potentially inappropriate medicines; PPOs = Potential prescribing omissions; OR = Odds Ratio. ^a^ Adjusted forgender, prior 12-month GP, deprivation, CHF, number of medication, NEADL activities of daily living. For age, non-Māori participants were born in 1925. Māori participants were born in 1920–1930. Age was adjusted for in all models. ^aa^Calculated as a percentage of non-Māori patients for whom hospitalisation data was known (*n* = 398). ^aaa^ Percentage total non-Māori population (n = 404)

#### Potential prescribing omissions (PPOs)

In adjusted models for Māori, the occurrence of PPOs was associated with greater risk of hospitalisation within the 12 month follow up period (51.7% with PPO hospitalised vs 24.3% without PPO hospitalised *p* = 0.001). There was also a difference for non-Māori (54.0% with PPO hospitalised vs 46.0% without PPO hospitalised) but this was not statistically significant *p* = 0.090. With regards to mortality, there was no association between PPOs and increased risk of mortality (*p* = 0.125 for Māori, and 0.160 for non-Māori) (Table 4).

## Discussion

This is the first study to describe the appropriateness of medicines prescribed to Māori and non-Māori octogenarians in New Zealand and the first study to our knowledge to prospectively identify a significant independent association between the occurrence of potential prescribing omissions (PPOs), and hospitalisation at 12 months follow-up.

Overall Māori had more PPOs, fewer PIMs and a lower overall quality of prescribing than non- Māori. Māori in New Zealand have documented ethnic related disparities in treatment and outcomes of cardiovascular disease [39]. In the current study, Māori had a higher prevalence of congestive heart failure, coronary artery disease, peripheral vascular disease and atrial fibrillation [40]. The few differences between PPOs for Māori and non-Māori included a higher rate of omission of Aspirin or Clopidogrel for vascular disease amongst Māori. While it is reassuring that there were few other disparities in potentially inappropriate medicines (PIMs) seen, this is evidence that treatment disparities persist into advanced age for Māori and are in need of further attention by New Zealand prescribers.

The occurrence of PPOs was more common than instances of PIMs in both Māori and Non-Māori (58 and 49% respectively). Those with PPOs were at an approximately 50% increased risk of hospitalisation during the 12 month follow up period. In contrast only 24 and 28% of Māori and Non-Māori respectively had at least one PIM and PIMS were not associated with either hospitalisation or mortality at 12 months follow-up. These findings have significant implications for clinical practice, particularly as recent strategies to improve medication related outcomes have focused on deprescribing [41, 42]. It is possible that uncontrolled confounding is part of this finding and the participants’ underlying multi-morbidity caused their hospitalisations. We advocate for balance in consideration of medication use in advanced age with the preservation of appropriate medication.

The reasons for these omissions are not clear, but may result from conservative prescribing in an effort to avoid polypharmacy, in itself is a risk factor for medication-related problems in older adults [43, 44]. It may also be as a result of prescribers’ consideration of the potential benefits and adverse effects within the expected lifespan of each patient. However, there is a considerable body of evidence to support the prescribing of medicines listed in START. For example, the cardiovascular benefit of treating hypertension for patients aged 75 years and older, is the prevention of stroke within 2 years [45]. Whilst the average age of the patients in this cohort was 82.3 (±2.6) and 84.6 (±0.53) years for Māori and Non-Māori respectively, at the time of recruitment to this study, each cohort’s life expectancy was approximately age 91 and 92 years respectively, based on life expectancy statistics estimates by the NZ Government [46]. Additionally, the functional assessment of the cohort demonstrated independence, rather than dependence. Consequently, conservative prescribing practices based on the patient’s age and potential for limited life expectancy should not be a consideration.

Additionally, when assessing the presence of potential prescribing omissions, patients’ co-morbidities and concurrently prescribed medicines were taken into consideration, and an indicated medicine was only designated as an omission if there was no obvious reason for its omission. We are therefore confident with the accuracy of our assessment. We were, however, unable to consider patients’ preferences when conducting this assessment. Increasingly, patients are encouraged to engage with prescribers regarding decisions about their medicines and to discuss their medication taking preferences with prescribers [47]. It is possible that some participants in this study opted not to take the medicines recommended. Irrespective of the reason for prescribing omissions, this study highlights the importance of ensuring omissions are minimised.

The absence of antidepressant medicines in the presence of moderate/ severe depression was the commonest omission overall, accounting for nearly 20% of all prescribing omissions. Depression is common in older people (particularly those with multiple comorbidities) and it is generally accepted that the under-treatment of depression in older adults can have a significant impact on morbidity and mortality, particularly cardiac mortality [48]. However, it is also widely acknowledge that the diagnosis and treatment of depression in older adults is particularly challenging [49]. Other analyses of New Zealand data (BRIGHT Trial, [50] DELLITE Trial), [51] a disparity between the use of antidepressants and the level of depressive symptoms, and that treatment was not always entirely appropriate [52]. We add to the significant debate about primary care management of depression with a picture of complexity in prescribing for depression for those in advanced age [53].

The omission of calcium and vitamin D_3_ supplement in patients with known osteoporosis was also notable. Whilst the evidence base for this particular criterion is well established,[54–56] recent concerns regarding the safety of calcium supplements, particularly regarding the occurrence of cardiovascular events, might explain the under prescribing of this medicine [57]. As concerns around the prescribing of calcium supplements originated in New Zealand, prescribers in New Zealand may be particularly cautious to its effects, influencing the noted omission.

While this study is prospective, causality cannot be proven as the potential for confounding health related factors not measured or adjusted for is high. We did however adjust for the main causes of hospitalisation and number of medications, increasing the likelihood that the identification of the omission of medications is a robust correlate of hospitalisation. In addition we have made multiple comparisons of the criteria between the ethnic groups. In presenting our results, we have taken the approach of Rothman [58] where he argues that it is better to describe all the significance tests performed, and allow the reader to reach a reasonable conclusion than to over adjust for Type I error which automatically increases the likelihood of a Type II error, therefore we did not apply a Bonferoni correction. Readers should be cautious in over interpretation of these differences.

Looking at the overall rates of potentially inappropriate prescribing, the rates noted in this study are lower than those noted in other studies using the STOPP/START criteria. Whilst there are no other studies that have specifically investigated potentially inappropriate prescribing using both the STOPP and START criteria in a community dwelling octogenarian cohort, Ubeda and colleagues [59] reported that, in an institutionalised cohort with a mean age of 84 years, the PIM rate using STOPP was 48%, (compared to 25 and 28% for Māori and non-Māori) and the PPO rate using START was 44% (compared 58 and 49% for Māori and non-Māor**i** respectively). In the current study, those who completed only the core interview were more likely to be dependent, suggesting a possible underestimation of the true prevalence of PIMs. Similarly, rates of inappropriate prescribing ascertained using a subset of STOPP was reportedly higher in a cross-sectional study conducted in the United Kingdom (33% for 81-85 years) than in the present study. [60] Perhaps prescribers for participants of the LiLACS NZ study have a more conservative approach.

It is possible to apply the STOPP criteria to large primary care databases to establish the occurrence of potentially inappropriate prescribing. [13,60] More routine use of the criteria may have clinical utility, for example, the recent ‘pill pruner’, have operationalised an abbreviated set of STOPP criteria [58]; further work will be needed for to easily use the START criteria. This paper suggests that work in now timely.

## Conclusions

Potential prescribing omissions (PPOs) were more common than potentially inappropriate medicines (PIMs) in both Māori and non-Māori patients and were associated with an increased risk of hospitalisation in Māori patients. This study highlights the importance of ensuring that all indicated medicines are prescribed, and that there is regular review of medicines prescribed. Further follow-up over a longer time period is necessary to determine the long-term effects of potentially inappropriate prescribing on mortality and further hospitalisation.

### Limitations

As not the entire patients’ clinical data were available, not all of the STOPP/START criteria were applied, which may have led to a slight underestimation of the occurrence of PIP. The follow-up period was only 12 months; a longer follow up period will allow the effects of PIP on mortality to be further investigated. The reasons for hospital admissions were not investigated; these could have been correlated with the PPOs.

The study may also be limited in generalizability by the regional boundaries and single country nature of the design. Further studies in broader groups need to confirm the association between PPOs and hospitalisation. Prevalence related to prescribing patterns would be expected to be region and country specific as guidelines and medication regulation vary nationally and regionally.

It is possible that some of this cohort may have had a limited life expectancy. This would involve a small number of patients, for whom some PPOs may have been over-estimated. It is also possible that there are factors we were unable to control for that may explain the differences identified between those who have PPOs and those without PPOs. Inferential findings must always be viewed with caution from observational research.

## Supplementary information

**Additional file 1: Figure S1.** The percentage of medicines prescribed per ATC heading for the total population, Māori and non-Māori participants.

**Additional file 2: Figure S2.** 12 month hospitalisation (a) and 12 month mortality (b) for LiLACS NZ participants according to presence of at least one PIM, one PPO or either a PIM or PPO (unadjusted analyses).

**Additional file 3.** Appendix 1. Potentially inappropriate medicines (PIMs) identified by the STOPP criteria for the entire patient group, for Māori and non-Māori patients.

**Additional file 4.** Appendix 2. Potential prescribing omissions (PPOs) identified by the START criteria for the entire patient group, for Māori and non-Māori patients.

## Data Availability

All data presented in the manuscript is supported by appropriate tables as supporting material. The authors do not wish to make the data available as it contains confidential patient information.

## Abbreviations

ATC: Anatomical Therapeutic Chemical
CHF: Congestive Heart Failure
EGFR: Estimated GFR
LiLACS NZ: Life and Living in Advanced Age: A Cohort Study in New Zealand
MDRD: Modification of Diet in Renal Disease
NEADL: Nottingham Extended Activities of Daily Living
NZDep: New Zealand Deprivation Index
PIMs: Potentially inappropriate medicines
PIP: Potentially inappropriate prescribing
PPOs: Potential prescribing omissions
PVD: Peripheral Vascular Disease
SPSS: Statistical Package for the Social Sciences
