# Supplementary material for: Quality of prescribing predicts hospitalisation in octogenarians: life and living in advanced age: a cohort study in New Zealand (LiLACS NZ)
Source: BMC Geriatr. 2019 Dec 19;19:357. doi: 10.1186/s12877-019-1305-x (PMC6921419; doi:10.1186/s12877-019-1305-x)
Supplement: Supplementary file 1 — Additional file 1: Figure S1. The percentage of medicines prescribed per ATC heading for the total population, Māori and non-Māori participants. [file 12877_2019_1305_MOESM1_ESM.docx]

Figure S1: The percentage of medicines prescribed per ATC heading for the total population, Māori and non-Māori participants
