# Supplementary material for: Quality of prescribing predicts hospitalisation in octogenarians: life and living in advanced age: a cohort study in New Zealand (LiLACS NZ)
Source: BMC Geriatr. 2019 Dec 19;19:357. doi: 10.1186/s12877-019-1305-x (PMC6921419; doi:10.1186/s12877-019-1305-x)
Supplement: Supplementary file 2 — Additional file 2: Figure S2. 12 month hospitalisation (a) and 12 month mortality (b) for LiLACS NZ participants according to presence of at least one PIM, one PPO or either a PIM or PPO (unadjusted analyses). [file 12877_2019_1305_MOESM2_ESM.docx]

(a)

(b)

Figure S2: 12 month hospitalisation (a) and 12 month mortality (b) for LiLACS NZ participants according to presence of at least one PIM, one PPO or either a PIM or PPO (unadjusted analyses)

PIMs= Potentially inappropriate medicines; PPOs= Potential prescribing omissions; * Significant difference p<0.05
