# Supplementary material for: Quality of prescribing predicts hospitalisation in octogenarians: life and living in advanced age: a cohort study in New Zealand (LiLACS NZ)
Source: BMC Geriatr. 2019 Dec 19;19:357. doi: 10.1186/s12877-019-1305-x (PMC6921419; doi:10.1186/s12877-019-1305-x)
Supplement: Supplementary file 3 — Additional file 3. Appendix 1. Potentially inappropriate medicines (PIMs) identified by the STOPP criteria for the entire patient group, for Māori and non-Māori patients. [file 12877_2019_1305_MOESM3_ESM.docx]

**Appendix 1: Potentially inappropriate medicines (PIMs) identified by the STOPP criteria for the entire patient group, for Māori and non-Māori patients.**

| **Cardiovascular System** | **Total (n; %)** | **Māori (n; %)** | **Non-Māori (n; %)** |
| --- | --- | --- | --- |
| Digoxin at a long-term dose > 125µg/day with impaired renal function | 1 (0.4) | 1 (1.1) | - |
| Loop diuretic as first-line monotherapy for hypertension | 1 (0.4) | 0 | 1 (0.6) |
| Thiazide diuretic with a history of gout | 10 (4.0) | 7 (7.7) | 3 (1.9) |
| Use of diltiazem or verapamil with NYHA Class III or IV heart failure | 16 (6.5) | 9 (10.0) | 7 (4.5) |
| Calcium channel blockers with chronic constipation | 8 (3.2) | 3 (3.3) | 5 (3.2) |
| Use of aspirin and warfarin in combination without histamine H_2_ receptor antagonist | 6 (2.4) | 1 (1.1) | 5 (3.2) |
| Dipyridamole as monotherapy for cardiovascular secondary prevention | 1 (0.4) | 1 (1.1) | - |
| Aspirin at dose > 150mg day | 16 (6.5) | 9 (10.0) | 7 (4.5) |
| Aspirin with no history of coronary, cerebral or PVD symptom or occlusive event | 20 (8.1) | 10 (11.1) | 10 (6.4) |
| Tricyclic antidepressants (TCA’s) with dementia | 1 (0.4) | - | 1 (0.6) |
| TCA’s with glaucoma | 1 (0.4) | - | 1 (0.6) |
| TCA’s with constipation | 4 (1.6) | - | 4 (2.5) |
| TCA’s with an opiate or calcium channel blocker | 11 (4.5) | 1 (1.1) | 10 (6.4) |
| Long-term (i.e. > 1 month), long-acting benzodiazepines e.g. chlordiazepoxide, fluazepam, nitrazepam, chlorazepate and benzodiazepines with long-acting metabolites | 4 (1.6) | 1 (1.1) | 3 (1.9) |
| Prolonged use (> 1 week) of first generation antihistamines | 4 (1.6) | 1 (1.1) | 3 (1.9) |
| **Gastro-intestinal System** |  |  |  |
| PPI for peptic ulcer disease at full therapeutic dosage for > 8 weeks | 42 (17.0) | 15 (16.6) | 27 (17.2) |
| **Respiratory System** |  |  |  |
| Systemic corticosteroids instead of inhaled corticosteroids for maintenance therapy in moderate-severe COPD | 5 (2.0) | 3 (3.3) | 2 (1.3) |
| **Musculoskeletal System** |  |  |  |
| NSAID with moderate-severe hypertension | 11 (4.5) | 1 (1.1) | 10 (6.4) |
| NSAID with heart failure | 5 (2.0) | 3 (3.3) | 2 (1.3) |
| Warfarin and NSAID together | 1 (0.4) | - | 1 (0.6) |
| NSAID with chronic renal failure | 4 (1.6) | 3 (3.3) | 1 (0.6) |
| Long-term corticosteroids (>3 months) as monotherapy for rheumatoid arthritis or osteoarthritis. | 5 (2.0) | 1 (1.1) | 4 (2.5) |
| Long-term NSAID or colchicine for chronic treatment of gout where there is no contraindication to allopurinol | 12 (4.9) | 8 (8.9) | 4 (2.5) |
| **Falls** |  |  |  |
| Benzodiazepines | 14 (5.7) | 3 (3.3) | 11 (7.0) |
| Neuroleptic drugs | 6 (2.4) | - | 6 (3.8) |
| First generation antihistamines | 6 (2.4) | 1 (1.1) | 5 (3.2) |
| Vasodilator drugs with persistent postural hypotension i.e. recurrent > 20mmHg drop in systolic blood pressure | 9 (3.6) | 2 (2.2) | 7 (4.5) |
| Long-term opiates in those with recurrent falls | 23 (9.3) | 6 (6.7) | 17 (10.8) |
| **Total instances of PIP identified** | **247** | **90** | **157** |

*Mann-Whitney U Test
