# Supplementary material for: Quality of prescribing predicts hospitalisation in octogenarians: life and living in advanced age: a cohort study in New Zealand (LiLACS NZ)
Source: BMC Geriatr. 2019 Dec 19;19:357. doi: 10.1186/s12877-019-1305-x (PMC6921419; doi:10.1186/s12877-019-1305-x)
Supplement: Supplementary file 4 — Additional file 4. Appendix 2. Potential prescribing omissions (PPOs) identified by the START criteria for the entire patient group, for Māori and non-Māori patients. [file 12877_2019_1305_MOESM4_ESM.docx]

**Appendix 2: Potential prescribing omissions (PPOs) identified by the START criteria for the entire patient group, for Māori and non-Māori patients.**

| **START CRITERIA** | **Total (n;%)** | **Māori (n; %)** | **Non-Māori (n; %)** |
| --- | --- | --- | --- |
| ***Cardiovascular System*** |  |  |  |
| Warfarin or aspirin in the presence of chronic atrial fibrillation | 21 (3.6) | 11 (4.0) | 10 (3.2) |
| Aspirin or clopidogrel with a documented history of atherosclerotic coronary, cerebral or peripheral vascular disease | 37 (6.3) | 25 (9.0) | 12 (3.8) |
| Antihypertensives where systolic blood pressure is > 160mmHg | 59 (10.0) | 15 (5.4) | 44 (14.1) |
| Statin therapy with a documented history of atherosclerotic coronary, cerebral or peripheral vascular disease | 53 (9.0) | 30 (10.8) | 23 (7.4) |
| ACE Inhibitor with congestive heart failure | 59 (10.0) | 27 (9.7) | 32 (10.3) |
| ACE Inhibitor & Acute myocardial infarction | 47 (8.0) | 19 (6.8) | 28 (9.0) |
| β blocker with chronic stable angina | 67 (11.4) | 30 (10.8) | 37 (11.9) |
| ***Central Nervous System*** |  |  |  |
| Antidepressants in the presence of moderate/severe depression | 117 (19.8) | 58 (20.9) | 59 (19.0) |
| ***Musculoskeletal System*** |  |  |  |
| Bisphosphonate in patients taking maintenance corticosteroid therapy | 27 (4.6) | 16 (5.8) | 11 (3.5) |
| Ca^2+^ & Vit D_3_ supplement in patients with known osteoporosis | 62 (10.5) | 24 (8.6) | 38 (12.2) |
| ***Endocrine System*** |  |  |  |
| ACE Inhibitor/ ARBs in diabetes with nephropathy | 6 (1.0) | 3 (1.1) | 3 (1.0) |
| Antiplatelet therapy in diabetes with co-existing major cardiovascular risk factors | 15 (2.5) | 8 (2.9) | 7 (2.2) |
| Statin therapy in diabetes mellitus if co-existing major cardiovascular risk symptoms present | 20 (3.4) | 12 (4.3) | 8 (2.6) |
| ***Total instances of PPOs*** | 590 | 278 | 312 |

*Mann-Whitney U Test
